# Preliminary isolation and *in vitro* antiyeast activity of active fraction from crude extract of *Gracilaria changii*

**DOI:** 10.4103/0253-7613.44155

**Published:** 2008-10

**Authors:** Sreenivasan Sasidharan, Ibrahim Darah, Mohd Kassim Mohd Jain Noordin

**Affiliations:** 1 School of Biological Sciences, Universiti Sains Malaysia, Pulau Pinang, Malaysia; 2 School of Chemical Sciences, Universiti Sains Malaysia, Pulau Pinang, Malaysia; 3 Department of Biotechnology, AIMST University, Kedah, Malaysia

**Keywords:** Antiyeast activity, *candida albicans*, *Gracilaria changii*, marine algae

## Abstract

**Objective:**

To isolate the active fraction from crude extract of Gracilaria changii and to determine its *in vitro* antifungal activity.

**Materials and Methods:**

The active fraction was isolated from the crude extract of *G. changii* by various purification procedures such as column chromatography, thin layer chromatography, bioauthograph etc. The *in vitro* antifungal activity (*Candida albicans*) of the active fraction (1.00, 0.50, and 0.25 mg/ml) was studied by disc diffusion method and the effect of the active fraction on the morphology of yeast was done by scanning electron microscope (SEM) studies.

**Results:**

An active fraction with remarkable antifungal activity was separated from the crude extract. The active fraction was effective as a fungicide against *C. albicans* and showed a dose-dependent antifungal activity. A Scanning Electron Microscope (SEM) study confirmed the fungicidal effect of *G. changii* active fraction on *C. albicans*, by changing the normal morphology of C. albicans.

**Conclusion:**

From *G. changii* crude extract, an active fraction with remarkable in vitro antifungal activity has been isolated.

## Introduction

Today, there is an increasing demand for biodiversity in the screening programs seeking therapeutic drugs from natural products. There is great interest being shown in marine organism, especially algae or seaweed. The ability of seaweed to produce secondary metabolite has been extensively documented.[1,2] Recently, chemists worldwide have paid attention to the potential of marine microorganisms (e.g. bacteria, fungi, blue green algae, dinoflagellates etc.), as alternative sources for the isolation of novel metabolites with interesting biological and pharmaceutical properties.[3–10]

Malaysia is endowed naturally with very rich algae life. Among the algae with therapeutic properties in Malaysia, the *Gracilaria changii* has yet to gain importance and popularity. The *G. changii* belongs to the family of Gracilariaciae and is found predominantly in the mangrove areas of Malaysia. It grows abundantly in hot, humid equatorial countries such as Malaysia and Thailand. Although at one time it was considered to be a foodstuff because the alga is edible, the economic importance of this alga is now beginning to attract the attention of researchers. Our previous studies showed that the extract of *G. changii* exhibited good antimicrobial activity against *C. albicans*.[11]

In the present study, the antiyeast active fraction was purified from the crude extract of *G. changii* by various purification procedures. In addition to an *in vitro* bioassay for antiyeast activity, the effect of the active fraction against the morphology of *C. albicans* cells by SEM studies was also assessed.

## Materials and Methods

### Algae sample

Fresh *G. changii* sample was collected from Pantai Morib, Selangor, Malaysia, in January 2003 and authenticated by Prof. Phang Siew Moi (Institute of Biological Sciences, Faculty of Science, University of Malaya, Malaysia).

### Extraction procedure

At the field, the fresh alga was rinsed with seawater, to remove debris and epiphytes, before bringing it to the laboratory. In the laboratory, the algae was further washed with fresh water and brushed with a soft brush before being sun dried. The sun dried algae was cut into small pieces. Approximately 100 g of dried algae was added to 400 ml of methanol and soaked for four days. Removal of the algae from solvents was done by filtration through cheesecloth and the filtrate was concentrated using a rotary evaporator (EYELA, Japan).

### Microorganism

*Candida albicans*(local isolate) was used as the test organism and was obtained from the laboratory stock culture. The yeast was cultured in Sabouraud Dextrose agar at 30°C for 24 h. Stock cultures were maintained at 4°C on the slopes of Tryptic soy broth (BBL, Cockeysville, MD) amended with 5 g/l Yeast extract (Oxoid, Nepean, ON) and 15 g/l Agar (BDH, Toronto, ON).

### Isolation of antiyeast substances from crude extract of G. changii

The crude extracts were purified by column chromatography. The open glass column (150 by 200 mm) was packed with Silica gel (Merck, Darmstadt, Germany, 0.063-0.200 mm). The column, loaded with crude extracts, was eluted with methanol and chloroform (50:50 [vol/vol]). Each fraction (10 ml) of the eluate was concentrated in the rotary evaporator. The antiyeast activity of each fraction against *C. albicans* was measured by the disk diffusion method.[12] The active fraction, which showed a high antiyeast activity, was further purified by preparative thin-layer chromatography (TLC) (silica gel 60 F_254_ [0.2 mm thick]; Merck). Thin-layer chromatography plates, loaded with crude extracts, were developed with a chloroform-methanol (85:15 [vol/vol]) solvent system. After the plate was air dried, a silica gel band which showed antiyeast activity against *C. albicans* at the position of R_f_ 0.63 was collected by scraping off the band, and eluted with methanol. The inhibition zones produced on the TLC plates were visualized by the bioautographic technique.[13]

### Antimicrobial activity

The antimicrobial activity of the extract of the active fraction was determined following the method described by Miles and Amyes,[12] with slight modifications:

### Disk diffusion technique

The test microbes were removed aseptically with an inoculating loop and transferred to a test tube containing 5.0 ml of sterile distilled water. Sufficient inoculum was added, until the turbidity equaled to 0.5 McFarland standards (bioMerieux, Marcy Petoile, France). One ml of the test tube suspension was added to 15-20 ml of Nutrient agar, before setting aside the seeded agar plate (9 cm in diameter) to solidify for 15 minutes. Three Whatman's filter paper No. 1 disks of 6 mm diameter were used to screen the antimicrobial activity. Each sterile disk was impregnated with 20 *µ*l of the fraction (corresponding to 1.00, 0.50, and 0.25 mg/ml active fraction); Miconazole nitrate (30 µg/ml, as positive control) and methanol (v/v) (as negative control) were placed on the surface of the seeded plates. The plates were placed at 4°C for two hours, followed by incubation at 37°C, for 24 h and examined for zones of growth inhibition, which were expressed in millimeters (mm). Each test was performed in triplicate and repeated twice.

### Effect of the active fraction on C. albicans cells by scanning electron microscopy study

One ml of the *C. albicans* cells suspension of the concentration of 1x10^6^ cells per ml was inoculated on a Sabouraud dextrose agar plate and then incubated at 30°C for six hours. Ten *µ*l of the active fraction, at a concentration of 1.00 mg per ml, was dropped onto the inoculated agar and further incubated for another 36 hours, at the same incubation temperature. A methanol treated culture was used as a control. A small block of yeast containing agar was withdrawn from the inoculated plate, at various time intervals (control, 12, 24 and 36 hours) and fixed for SEM.[14]

### Statistical analysis

All the assays were carried out in triplicate. The results are expressed as mean values ± standard deviation (SD). The differences between the fractions are analyzed using the one-way analysis of variance (ANOVA), followed by Tukey's HSD test with α = 0.05. The difference between means was considered significant when *P* was <0.05. This analysis was carried out using SPSS 12.0 for Windows programmer.

## Result and Discussion

The column chromatography was run by the above method and it yielded 10 fractions. The antiyeast activity of each fraction against *C. albicans* was measured by the disk diffusion method and the third fraction showed a high antiyeast activity [Table 1]. Further purification was done by the preparative thin-layer chromatography, to identify the active fraction by bioautographic technique. The thin layer chromatography eluant system that was developed (methanol : chloroform, 15 : 85), separated compounds over a wide range of R_f_ values. The inhibition zones produced by TLC bioautographic technique, showed the band with antiyeast activity against *C. albicans* at the position of R_f_ 0.63. Bioautography of TLC chromatograms worked well with *C. albicans* in this study.[13] The band, which showed antiyeast activity was also viewed under UV and was blue fluorescent. Subsequently, the active fraction was collected by scraping off the band and then redissolved in methanol for the antimicrobial activity and SEM studies. The active fraction, therefore, holds the most promise for further work, as far as the antimicrobial components are concerned.

**Table 1:** Antimicrobial activity (Zone of inhibitiona)^a^ of fractions from *Gracilaria changii* compared to commercial antibiotic Miconazole nitrate

*Fraction*	*Zone of inhibition (mm)^b^*
Miconazole nitrate	22.00 + 0.45
Methanol	−
Fraction 1	−
Fraction 2	18.00 + 0.52
Fraction 3	20.00 + 0.32
Fraction 4	17.00 + 0.55
Fraction 5	15.00 + 0.45
Fraction 6	15.00 + 0.51
Fraction 7	10.00 + 0.43
Fraction 8	13.00 + 0.52
Fraction 9	10.00 + 0.39
Fraction 10	−

a Disk diffusion technique

b The values (mean ± SD; *P*<0.05) are diameter of zone of inhibition at 1.0 mg/ml of fractions and 30 µg/ml Miconazole nitratẽ no zone of inhibition

The active fraction, showing a dose-dependent antimicrobial activity against the yeast tested with the zones of inhibition, ranged from 12 to 20 mm. The zone of clearance produced by the commercial antibiotic (Miconazole nitrate) disk was larger than those produced by the active fraction disk [Table 2]. The solvent-only negative control disk produced no zone of clearance [Table 2].

**Table 2:** Dose-dependent antimicrobial activity (Zone of inhibitiona)^a^ of the active fractionfrom *Gracilaria changii*

*Microorganism*	*Dose*	*Zone of Inhibition (mm)^b^*
*Candida albicans*	Methanol	
	0.0	−
	Miconazole nitrate	
	30.00 µg/ml	22.00 ± 0.46
	Active fraction	
	0.25 mg/ml	12.00 ± 0.33
	0.50 mg/ml	16.00 ± 0.42
	1.00 mg/ml	20.00 ± 0.39

a Disk diffusion technique

b The values (mean ± SD; *P*<0.05) are diameter of zone of inhibition (n = 3), no zone of inhibition

**Figure 1:**
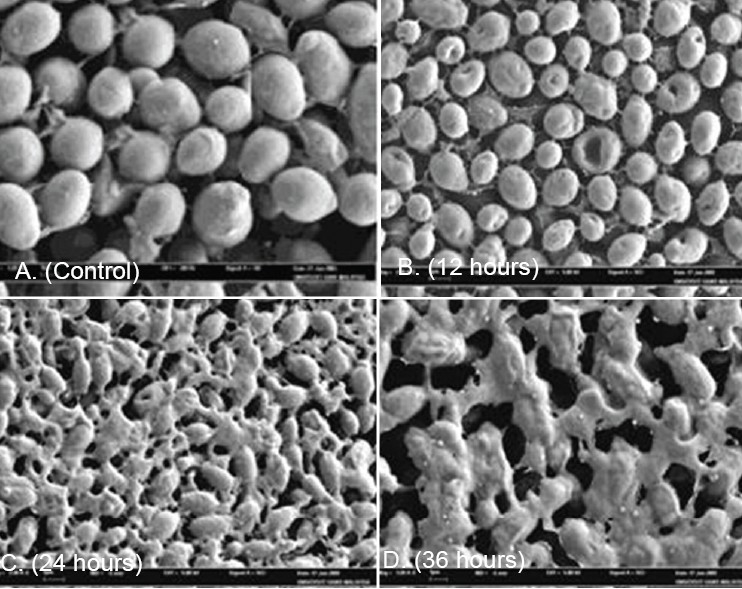
Scanning electron microscope photomicrograph of the untreated (A) and extract-treated (B, C and D) cells of *Candida albicans*

Figure 1 shows the SEM photomicrographs of the untreated and active fraction treated cells of *C. albicans*, at various times of exposure to the active fraction of *G. changii*. Untreated cells [Figure 1A] showed many oval and smooth cells in appearance and some at a budding stage. After 12 hours of exposure [Figure 1B], a significant effect of the fraction was observed. The cells treated for 12 hours showed rough cell appearance, when compared to the untreated control cells. The cells treated for 24 hours [Figure 1C] and 36 hours [Figure 1D] showed severe alterations and completely collapsed and lysed of yeast cells. It was believed that at this stage, the cells had lost their metabolic functions completely. The wide strong antimicrobial activity of active fraction may provide clinically useful leads. Active fraction is being further purified for chemical characterization and structural elucidation.

Further purification of the active fraction and *in vivo* evaluation of antiyeast activity along with toxicity studies of the active fraction are, therefore, suggested for further studies, on the basis of the present study.
